# Peptide-Based Nanomaterials for Tumor Immunotherapy

**DOI:** 10.3390/molecules26010132

**Published:** 2020-12-30

**Authors:** Lingyun Li, Bing Ma, Weizhi Wang

**Affiliations:** School of Chemistry and Chemical Engineering, Beijing Institute of Technology, Beijing 100081, China; 3220191170@bit.edu.cn

**Keywords:** peptide-based nanomaterials, immune checkpoint blockade, peptide self-assembly, peptide screening

## Abstract

With the increasing understanding of tumor immune circulation mechanisms, tumor immunotherapy including immune checkpoint blockade has become a research hotspot, which requires the development of more accurate and more efficient drugs with fewer side effects. In line with this requirement, peptides with good biocompatibility, targeting, and specificity become favorable theranostic reagents, and a series of promising candidates for tumor immunotherapy based on peptides have been developed. Additionally, the advantages of nanomaterials as drug carriers such as higher affinity have been demonstrated, providing possibilities of combination therapy. In this review, we summarize the development of peptide-based nanomaterials in tumor immunotherapy from the two aspects of functionalization and self-assembly. Furthermore, new methods for peptide screening, especially machine-learning-related strategies, is also a topic we were interested in, as this forms the basis for the construction of peptide-based platforms. Peptides provide broad prospects for tumor immunotherapy and we hope that this summary can provide insight into possible avenues for future exploration.

## 1. Introduction

Precision medicine has emerged as a new topic in the treatment of serious diseases such as cancer and intracranial disease. Researchers hope to use this “precision strike” method to make the treatment of these diseases more efficient. Targeting the designated part and delivering specific biological information are the keys to this strategy, for which molecules like peptides and antibodies, with their characteristics of targeting and specificity, have become promising candidates. Peptides are composed of various amino acids connected with amide bonds. The combination and arrangement of amino acids with different side groups enables peptides to encode huge amounts of biological information, and gives peptides different structures and multiple biological functions. Compared to antibodies, peptides are easier to synthesize and chemically modify, with better bioavailability, better biocompatibility, and lower immunogenicity, which make them competitive options [1,2]. Although a single peptide might become a promising candidate due to its superior biochemical properties, the potential of such peptides in diagnosis and treatment is much greater: peptide-based nanomaterials are taking the limelight. Firstly, peptides can be conjugated with nanoparticles such as liposomes to give them specific targeting ability, which can help them to be rapidly taken up by specific cells [3,4,5]. Secondly, as combination therapy has become the general trend, the introduction of nanomaterials has greatly expanded the possibilities of drug-design concepts, making it possible for different therapies to play a synergistic role regardless of the macroscopic cooperation between physical and chemical methods or the co-inhibition of different pathways of disease or even related proteins. Furthermore, peptides can self-assemble and arrange into nanofibers, nanoparticles, or nanotubes due to their own aggregation ability or when modified by aggregation molecules. These aggregates can then be used as drug carriers with enhanced targeting and penetration [6]. In a word, peptide-based nanomaterials provide a solid foundation and a broad platform for precision medicine, and are expected to lead to a breakthrough in diagnosis and treatment.

Cancer is one of the most serious diseases. Compared to surgery, chemotherapy, and targeted drugs, immunotherapy presents a promising strategy for curing cancer, which has been called “the third anticancer revolution”. Tumor immunotherapy improves the body’s own immunity as well as having the ability to kill tumors by activating the immune system, which has fewer side effects and longer tumor treatment effect than other treatment modalities. Immunotherapy can not only provide new methods for some cancers that are not treatable by chemotherapy or radiotherapy, but can also be used as an auxiliary to obtain better results through combination therapy [7]. Based on several links in the tumor immune cycle, screening appropriate molecules, including peptides, to enhance the immunity of T cells is a promising overall design idea. For instance, antigens released by tumor cells and presented to T cells are an important link in activating immunity, and a variety of peptide-based antigens have been developed as tumor vaccines [8,9,10]. In addition, there is a series of proteins expressed on immune and immune-related cells called immune checkpoints, which play an important role in immune regulation. These immune checkpoints can inhibit the immune response by inhibiting the activity and proliferation of immune cells upon binding to receptors on tumor cells [11]. Based on this fact, developing molecules that can target immune checkpoints or their ligands to block these pathways through competitive binding has become a mainstream strategy for tumor immunotherapy. Among the many immune checkpoints, programmed cell death 1 (PD-1) and its ligand programmed cell death ligand 1 (PD-L1) have become the “star molecules” due to their high expression levels in a variety of tumor cells. Many types of inhibitors have been developed to block the PD-1–PD-L1 pathway, including peptides [12]. Since Aurigene Discovery Technologies and Pierre Fabre developed the first anti-PD-1 peptide in 2014, many PD-1–PD-L1 pathway blocking peptides have been reported [13]. Not limited only to targeting, the development of peptide-based inhibitors has made great progress from structure to function, including cyclization, isolated from natural ligands, and induction of PD-L1 internalization [14,15,16,17]. However, high chance of relapse and few beneficiaries are the foremost obstacles to their translation to the clinical setting, which require the intervention of nanomaterials to break the deadlock.

Screening out suitable peptides with high affinity and stability is the basis of constructing a peptide-based drug platform, so developing effective screening methods is particularly critical. Traditional screening methods such as combinatorial chemical library screening and phage display technology are inefficient, clumsy, and isolated from the body environment. From a development view, the development of fast, multiparameter, in situ or bionic screening technologies is an inevitable trend. So far, many high-throughput chips and degenerate peptide libraries have been developed with the aim of improving the screening speed, which, combined with mass spectrometry and SPR (Surface plasmon resonance, a biosensing technology used to analyze the interaction between ligand and analyte), can capture more parameters in real time, as many reviews have summarized [18,19,20]. In fact, the prosperity of computer science has brought dramatic changes to research in many scientific fields. Researchers hope to use this intelligent, efficient, and precise strategy to perform large-scale statistical data analysis, and to find regular patterns to make predictions through calculations and analysis, which is highly compatible with the concept of screening high-quality peptides. In this review, we focus on new screening methods improved by advanced technologies, especially computer-based methods such as machine learning. Additionally, we give a comprehensive overview of peptide-based nanomaterials for tumor immunotherapy especially in immune checkpoint blockade, which has rarely been reported previously. Herein, we mainly focus on the design principles of these peptide-based nanomaterials.

## 2. Peptide-Functionalized Nanomaterials

**Figure 1:**
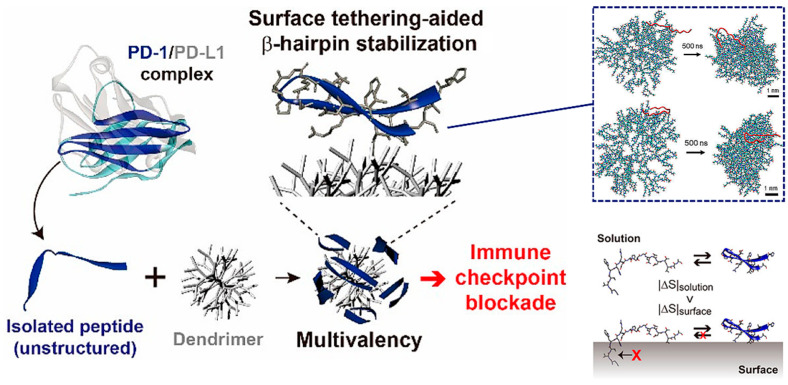
The peptide−dendrimer conjugate (PDC) platform has an enhanced immune checkpoint inhibitory effect due to the multivalent binding effect, and stabilizes the β-hairpin structure of the peptide via intermolecular forces and the excluded volume effect. Reproduced from Reference [23] with permission. Copyright: 2020 American Chemical Society.

Based on the above-mentioned advantages, many peptides for tumor immunotherapy have been identified, but few have been translated to the clinical context, which means that the free peptides have many intrinsic drawbacks such as low affinity and poor stability that limit their further development. To overcome these limitations, in addition to modifying the structure of the peptide itself, introducing nanomaterials with various properties is a promising strategy [21]. Multimerizing and conformational stabilizing are effective ways to enhance the peptide–protein interactions, which can be achieved by introducing appropriate macromolecular scaffolds. For instance, Maass et al. used phage display technology to achieve direct evolution of the cystine knot peptide MCoTI-II, which can bind to cytotoxic-T-lymphocyte-associated antigen 4 (CTLA-4, the first immune checkpoint recognized), an inhibitory receptor expressed by T lymphocytes. Fusion to antibody Fc domain or the oligomerization domain of C4b binding protein then resulted in oligovalent variants with greatly improved affinity, up to 400-fold [22]. Jeong et al. are committed to research on the conjugation of peptides with nonbiological materials. They developed a peptide−dendrimer conjugate (PDC) platform to block the PD-1 pathway, which multivalently conjugated the β-hairpin peptide, isolated from an engineered PD-1 protein, to the dendrimer surfaces to improve the affinity through cooperative interactions with multiple PD-L1 proteins on tumor cells (Figure 1) [23]. Additionally, the conjugation on the dendrimer surface assisted peptides in folding into their native structure, a β-hairpin, due to the excluded volume effect and peptide−dendrimer interactions, for which they had good stability. All these works taking advantage of dendrimers have given the PDC platform a stronger blocking ability than free peptides, and provide a new approach for peptide-based immune checkpoint blockade. 

**Figure 2:**
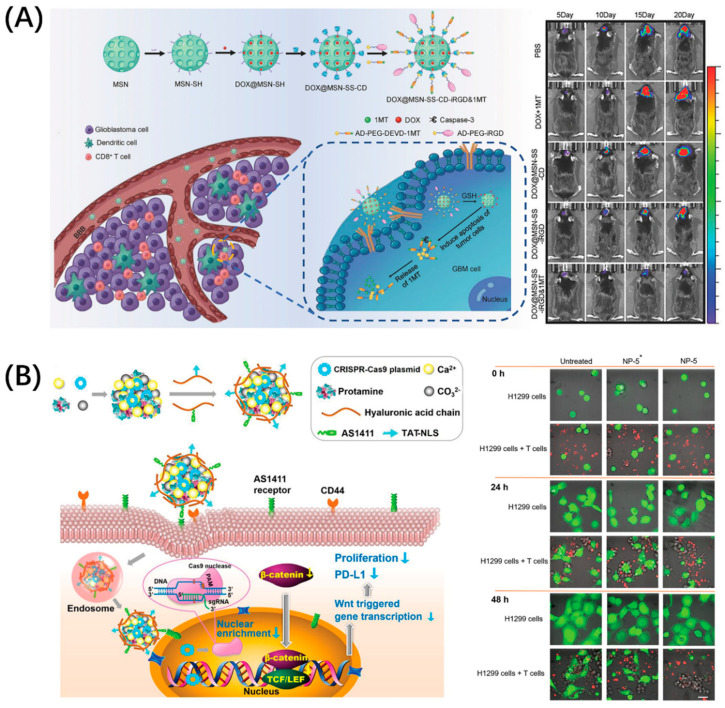
Peptide-functionalized nanomaterials for immune checkpoint blockade. (**A**) iRGD-modified nanoparticles, DOX@MSN-SS-iRGD&1MT, simultaneously delivered chemotherapeutic agent DOX and immune checkpoint inhibitor 1MT into orthotopic glioma, which exerted a superior antitumor effect in vivo. Reproduced from Reference [28] with permission. Copyright: 2018 WILEY-VCH Verlag GmbH & Co. KGaA, Weinheim. (**B**) Peptide TAT-NLS with cell-penetration and nuclear-transport functions mediated CRISPR–Cas9 (gene editing technology, which modifies specific DNA of targeted genes) plasmid delivery for β-catenin knockout and blocked the PD-1/PD-L1 pathway to improve the antitumor immune response of immune cells. Reproduced from Reference [29] with permission. Copyright: 2020 WILEY-VCH Verlag GmbH & Co. KGaA, Weinheim.

The PD-1/PD-L1 pathway has become the most intensively researched immune checkpoint due to its widespread presence in a variety of tumors. In fact, in addition to PD-L1, PD-1 has another known ligand, programmed cell death ligand 2 (PD-L2), which gives drugs blocking PD-1 a better immune stimulation effect [24]. Moreover, PD-L1 has become an important marker for tumor diagnosis; PD-L1-targeting molecules can not only be used for blocking, but are also potential candidates for tumor diagnosis [25,26,27]. Developing drugs targeting the PD-1/PD-L1 pathway is an important strategy for tumor immunodiagnosis and treatment. The enrichment of drugs in the lesion is the key to enhancing the therapeutic effect and reduce toxicity. Many drug carriers with good biological properties have been developed to solve these problems, which are not specific. Peptides with good targeting and penetrability can be conjugated with these nanocarriers, which can guide the drug-delivery system to reach the tumor efficiently and achieve precise killing. Zhang et al. combined brain tumor treatment with immunotherapy by using the classic blood–brain-barrier-penetrating peptide iRGD to build an immune checkpoint inhibitor delivery platform, DOX@MSN-SS-iRGD&1MT (Figure 2A) [28]. This platform comprised mesoporous silica nanoparticles loaded with DOX, which were then combined with Asp-Glu-Val-Asp (DEVD) to connect to 1-methyltryptophan (1MT), a PD-L1 inhibitor, and finally modified by iRGD. Consequently, this platform can upregulate antitumor cytokines while downregulating protumor protein expression, which produced the advantage of good brain tumor accumulation. Even more exciting is that the multiple structures and functions of peptides allow them to be combined with advanced technologies such as CRISPR–Cas9, for which He won the Nobel Prize in Chemistry in 2020. He et al. designed a peptide-functionalized genome-editing system, which was intended to achieve β-catenin knockout by delivering a CRISPR–Cas9 plasmid to the tumor nucleus based on the cell-penetrating and nuclear-translocation function of TAT-NLS (Figure 2B) [29]. This system enhanced the antitumor immune responses of immune cells by not only suppressing the Wnt/β-catenin pathway (a pathway related to nuclear translocation), but also notably downregulating the expressions of some immune checkpoint proteins like PD-L1 and CD47 (integrin-associated protein, inhibits the phagocytosis of tumor cells through binding to signal-regulatory protein α on macrophages) and by suppressing the oncogene Myc, which can bind to the promoters of *PD-L1* and *CD47* genes. In general, compound drug-delivery platforms have become a trend. Superior materials are selected according to needs, and rational and effective design can make the treatment more efficient while providing more diversified treatment strategies.

**Figure 3:**
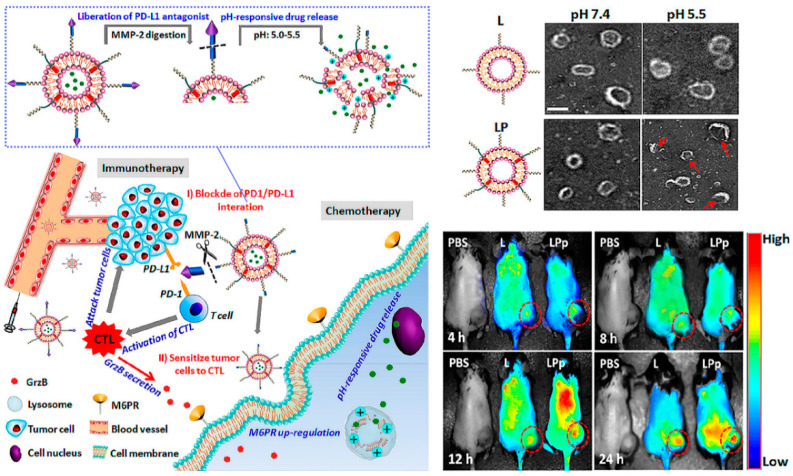
Combination immunotherapy with immune checkpoint blockade. pH and enzyme dual-responsive polymer–liposomes (LPDp) grafted with anti-PD-L1 peptide and encapsulating low-dose doxorubicin (DOX) for combination of cancer immunotherapy and chemotherapy. As shown in theTEMimages of L or LP with 2 mol % of pH-responsive polymer in PBS buffer solution, under the weakly acidic conditions of the tumor microenvironment, LP can respond to smaller nanospheres in order to avoid clearance by the immune system. And in vivo imaging has been proven it enriched at the tumor site with higher intensity. Reproduced from Reference [32] with permission. Copyright: 2019 American Chemical Society.

It is of note that peptides targeting immune checkpoints can not only guide drug delivery, but also block immune pathways, which can help researchers to cleverly construct a combination therapy platform. For instance, Wang et al. reported a synergistic liposomal drug-delivery system, SELS, functionalized with both an anti-ER antibody that identified ER-positive breast cancer tumor specifically, and the immune blocking peptide SP (sequence: KLEIITGERTLETVECTYNGG), which targets CD47 to enhance the immune response by reducing phagocytosis of the nanoparticles through macrophages (Figure 3) [30]. This synergistic liposome with enhanced targeting ability increased the tumor uptake and resisted immune circulation clearance simultaneously, which improved tumor imaging and therapeutic performance in living systems. As a result, the synergistic liposomal drug-delivery system proved that peptides have the ability to make up for the defects of function-limited drug carriers.

Conversely, from the perspective of immune checkpoint blockade, few beneficiaries and ease of relapse are huge challenges. Combination therapy with various tumor treatments is proving to be an effective strategy. Nanocarriers functionalized by peptides are create a “magic pocket”, of which the structure and biological and chemistry information loaded are everchanging and evolving, which provides unlimited possibilities for tumor immunotherapy [31]. Liu et al. designed a pH-sensitive liposome loaded with a low dose DOX to sensitize tumor cells to cytotoxic T lymphocytes (CTLs) by inducing overexpression of mannose-6-phosphate receptor (M6PR, regulates cell apoptosis) on tumor cell membranes, and conjugated with the hydrolysis-resistant D-peptide (NYSKPTDRQYHF) PD-L1 inhibitor to the surface, which can be released by matrix metalloproteinase (MMP) enzyme digestion [32]. In brief, this combination therapy platform can be divided into two parts: the peptide-based PD-L1-pathway inhibitor, and the immune boosting auxiliary system through chemotherapy. Consequently, this immune checkpoint blockade–auxiliary blockade system exhibited enhanced antitumor effects in vivo, and proved that nanomaterials create the possibility for immune combination therapy.

## 3. Self-Assembling Peptide Nanomaterials

**Figure 4:**
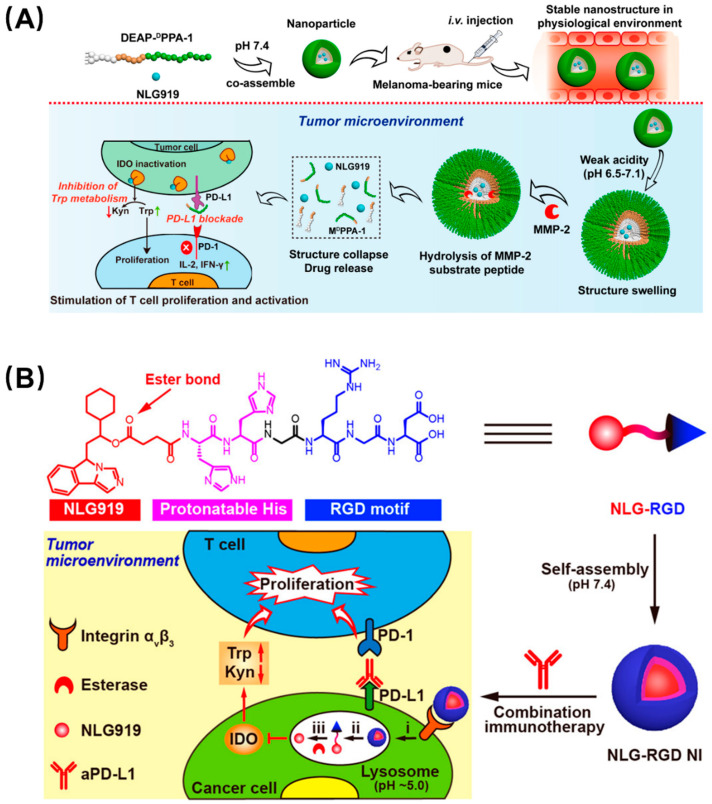
Synergistic nanoparticles blocked both PD-L1 and IDO to enhance tumor immunity. (**A**) DEAP-^D^PPA-1 and NLG919 coassembled into a nanoparticle which swelled in response to tumor acidity and dissociated through MMP-2 cleavage. Thereafter, NLG919 and ^D^PPA-1 were released to target IDO and PD-L1, respectively. Reproduced from Reference [37] with permission. Copyright: 2018 American Chemical Society. (**B**) Modularly designed, self-assembled peptide–drug conjugate NLG-RGD augmented antitumor immunity of PD-L1 checkpoint blockade by targeting IDO. It was self-assembled from a modularly designed peptide−drug conjugate containing a hydrophilic targeting motif RGD, two protonatable histidines, and an ester-bond-linked hydrophobic IDO inhibitor. It achieved potent and persistent inhibition of intratumoral IDO activity with a reduced systemic toxicity. Reproduced from Reference [38] with permission. Copyright: 2020 American Chemical Society.

Like the phenomenon of protein self-assembly that exists widely in nature, peptides can also self-assemble by using hydrogen bonding, electrostatic interaction, hydrophobic interaction, and π–π stacking between peptide bonds and amino acid residues, which is orderly, controllable, and stable [33]. Peptide self-assembly is generally based on their secondary structure or hydrophobicity. The common secondary structures in the peptide self-assembly process mainly include the α-helix, β-sheet, and β-hairpin, which make peptides assemble into fibers, nanotubes, and other supramolecular structure through the action of noncovalent bonding forces. In addition, hydrophobic interaction can drive peptides to form into vesicles [34,35]. Self-assembled peptides can not only provide increased affinity and specificity due to multivalent recognition, but drugs can be loaded into their cavities, and they can control the release of drugs as carriers [36]. For tumor immunotherapy, peptide-based self-assembling nanomaterials have many advantages: firstly, self-assembled peptide inhibitors can improve the efficiency of immune checkpoint blocking through enhanced affinity as well as spatial hindrance; secondly, whether immune checkpoint blockade or vaccine, it has been proven that combination therapy or addition of a vaccine adjuvant can alleviate the problems of poor immunity, ease of relapse, and small audience, which can be addressed by peptide-based self-assembling nanocarriers. According to this concept, PD-L1 and indoleamine 2,3-dioxygenase (IDO) were selected as a pair of promising immune blockade combination sites. IDO is a cytosolic tryptophan-catabolizing enzyme that can convert the essential amino acid L-tryptophan (Trp) to kynurenine (Kyn), which is constitutively expressed in many tissues and would induce immune tolerance. Combination therapy of PD-L1 and IDO is considered to have great potential in reducing the immune tolerance of PD-L1 inhibitors and is the subject of active clinical research. Hoping to apply the superior biological properties of self-assembled peptides to tumor immune combination therapy, Nie et al. designed a series of peptide-assembling nanoparticles with an enhanced immune checkpoint suppression effect based on the synergy of PD-L1 and IDO (Figure 4A) [37]. They conjugated an anti-PD-L1 short peptide, ^D^PPA-1, and a matrix metalloproteinase-2 (MMP-2) substrate, 3-diethylaminopropyl isothiocyanate (DEAP), to an amphiphilic molecule, which can coassemble into a particle with the IDO inhibitor NLG919. This nanoparticle swelled in a weakly acidic tumor microenvironment and was further cleaved by the MMP-2 enzyme to release the two synergistic inhibitors to promote the activation of T lymphocytes. Furthermore, a self-assembled nanoparticle targeting IDO alone has also been reported (Figure 4B) [38]. This rationally designed single-peptide-based molecule has four parts, including the hydrophilic peptide RGD to identify the tumor cells, two histidines as a pH-recognizable moiety to drive disassembly in the lysosome, an ester bond to achieve controlled drug release upon esterase treatment, and a hydrophobic unit, NLG919, which can inhibit IDO activity. The assembled nanoparticles can be cleaved by weak acids and MMP-2 in the tumor microenvironment to release drugs, and can thus effectively and continuously inhibit IDO activity in tumors and greatly enhance the inhibition of PD-L1 in vivo.

**Figure 5:**
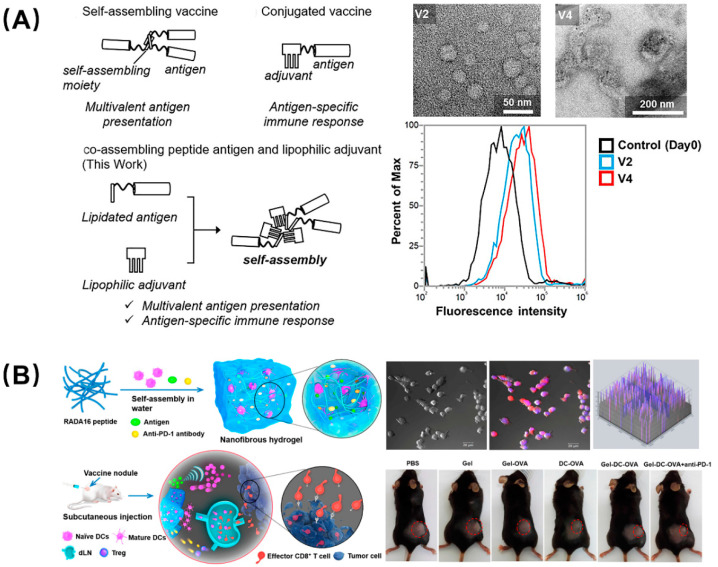
Peptide-based self-assembling vaccines for tumor immunotherapy. (**A**) Self-adjuvanting vaccines composed by coassembling a lipidated antigenic peptide and lipophilic adjuvants. Among them, vaccines V2 and V4 showed the best therapeutic ability, and flow cytometry showed that they still had high-strength binding to the BT-474 cells (high HER2 expression) on the 49th day. Reproduced from Reference [39] with permission. Copyright: 2020 Wiley-VCH GmbH. (**B**) Engineering dendritic-cell-based vaccines and PD-1 blockade in self-assembled peptide nanofibrous hydrogel to amplify antitumor T-cell immunity. DCs cultured in the hydrogel were used as exogenous vaccines or differentiated into endogenous vaccines in vivo and, after being injected into mice, the tumor volume was greatly reduced. Reproduced from Reference [40] with permission. Copyright: 2018 American Chemical Society.

There is a consensus that adjuvants can nonspecifically enhance the body’s specific immune response to antigens and assist vaccines in activating the immune system. Developing platforms capable of delivering antigen and adjuvant simultaneously allow any carrier-induced immunosuppression to be avoided and reduce inflammation side effects. For instance, Aiga et al. reported a coassembling vaccine composed of an antigenic CH401 peptide (YQDTILWKDIFHKNNQLALT) derived from human epidermal growth factor receptor 2 (HER2, a breast cancer tumor marker) and three types of lipophilic adjuvant (Figure 5A) [39]. This self-adjuvanting vaccine can assemble to fiber and particles, and induced enhanced immune response due to multivalent effects as well as the synergy of antigen and adjuvant; it is was considered to be an efficient and practical self-adjuvant vaccine candidate. Compared with tumor vaccines, immune cell adoptive therapy has attracted attention due to its precise targeting, speed, and efficiency, but it still has many flaws, such as low cell viability, transient duration of transplanted DCs at the vaccination site, and the lack of recruitment of host dendritic cells (DCs). Yang et al. designed a peptide nanofibrous hydrogel loaded with DCs, tumor antigen, and PD-L1 inhibitors to address these concerns (Figure 5B) [40]. The DCs in the hydrogel mature as a foreign vaccine by absorbing the antigen, and the sustained release of the antigen recruits the host DCs to form an autologous vaccine, which can provoke and amplify the antigen-specific T-cell immunity. In parallel, the PD-L1 inhibitors encapsulated in the hydrogel can block the immune checkpoint to alleviate the tumor immunosuppression. This combination therapy, which combined immune cells, antigens, and immune checkpoint inhibitors, provides a unique perspective and broad prospects for tumor immunotherapy.

**Figure 6:**
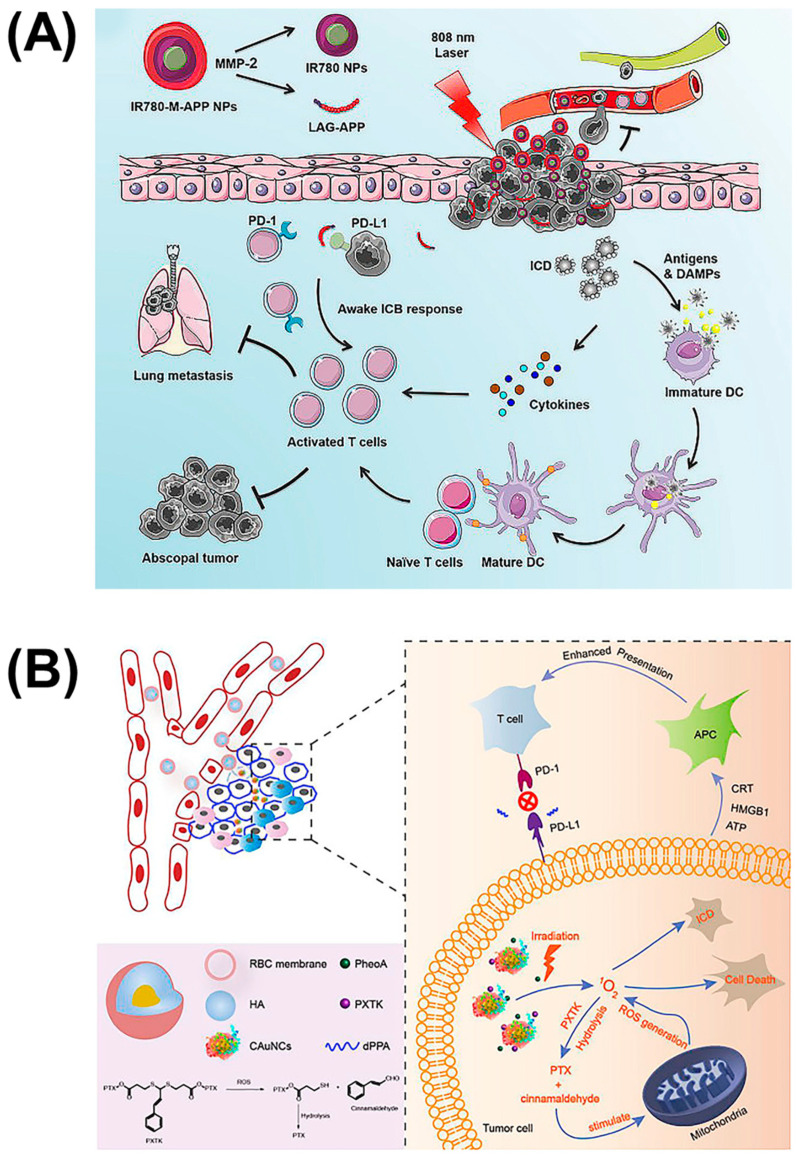
Self-assembled nanoparticles of reduced size combined with PDT to inhibit tumor metastasis. (**A**) Nanoparticles composed of photosensitizer IR780 and anti-PD-L1 peptide ^D^PPA-1, which can be triggered to transition to a smaller size by MMP-2 for advanced cancers. Reproduced from Reference [42] with permission. Copyright: 2020 Elsevier. (**B**) Hyaluronidase-responsive size-reducible biomimetic nanoparticles (mCAuNCs@HA) enhanced anti-metastasis efficacy through combination with PDT. Reproduced from Reference [43] with permission. Copyright: 2018 Elsevier.

Achieving high-intensity precision strikes is the key to immunotherapy. Conventional immunotherapy methods cannot solve the problem of serious side effects and tumor metastasis due to the low concentration of drugs at the lesion site. In addition to multisite targeting and the cooperation of multiple pathways to stimulate immunity, combination with physical therapies such as photodynamic therapy (PDT) provides a new strategy. PDT usually uses a specific wavelength to irradiate the tumor site, which can activate a photosensitive drug, selectively gather in the tumor tissue, and transfer energy to the surrounding oxygen, generate reactive oxygen species (ROS) to trigger oxidative reactions, then produce cytotoxicity to kill the tumor. In attempts to improve the moderate immunity of immune checkpoint blockade caused by the tumor microenvironment, combination therapy with PDT has attracted considerable attention. In addition to applying an IDO-suppression strategy to brain tumor treatment, Zhang et al. also combined it with photosensitizers [41]. They synthesized a chimeric peptide, PpIX-1MT, which integrated photosensitizer PpIX with IDO inhibitor 1MT via the caspase-responsive peptide sequence DEVD. This molecule can self-assemble into nanoparticles with the hydrophilic effect of PEG, and releases ROS upon 630 nm light irradiation, which can induce apoptosis of cancer cells and promote the expressions of caspase-3 (a splicing enzyme related to apoptosis) to release 1MT through the clearage. Similarly, Wang et al. coupled the photosensitizer IR780 to the anti-PD-L1 peptide ^D^PPA-1, which can be cleaved by MMP-2 to exert an immune checkpoint blocking function (Figure 6A) [42]. The difference is that this IR780-M-APP platform cam self-assemble into nanoparticles without any other excipient, which ensures the content of APP. Moreover, this nanoparticle can also be triggered by the MPP-2 enzyme to transform to a smaller size, which is able to significantly increase tumor accumulation and eradicate metastatic tumors. To reduce the impact of size on nanoparticle drug delivery, Qiu et al. designed sequentially responsive biomimetic nanoparticles with an optimal size, mCAuNCs@HA, the size of which can be reduced in response to hyaluronidase in order to penetrate deeply into tumor tissue (Figure 6B) [43]. A natural long-circulation delivery vehicle, the RBC membrane, was then coated on the outside to prevent the therapeutic from macrophage uptake without affecting the degradation process. It is worth noting that the anti-PD-L1 peptide encapsulated by this variable-size nanoparticle was also the D-type hydrolysis-resistant peptide, ^D^PPA-1, a promising candidate as a PD-L1 inhibitor for its superior stability in the blood circulation [14]. Based on ^D^PPA-1, Peng et al. introduced the 27 amino acid peptide CF27, coassembled with crosslinker *N*′-bis(acryloyl)cystamine (BISS) to form a crosslinking network structure to further coat the drug-dye nanoparticles, assembled by chemodrug DTX and induced by near infrared dyes IR820 as carriers [44]. Firstly, this nanoparticle can be cracked by MMP-2 to release the PD-L1 inhibitor, and then BISS can respond to glutathione (GSH) inside the cells to release IR820. Thereafter, this MMP/GSH dual-responsive nanoparticle can be used as a photothermal conversion agent for photothermal therapy while blocking the PD-1/PD-L1 pathway, which makes it a promising candidate for tumor combinatorial immunotherapy. In general, all these platforms combining immune checkpoint inhibitors and photosensitizers have proven that it is an efficient combinatorial therapy, which can efficiently eliminate the primary tumor and inhibit distal tumors or alleviate metastasis.

## 4. New Methods for Peptide Screening and Construction

**Figure 7:**
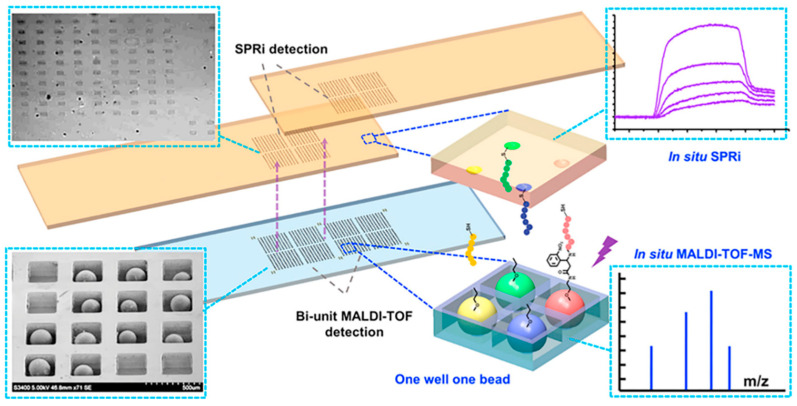
New methods for improving the speed of peptide screening. A high-throughput chip for screening, in which peptide beads were trapped in the microwell array for MALDI-TOF-MS detection and peptides were photocleaved in situ and imprinted onto the SPRi (surface plasmon resonance analysis imaging system) chips for SPRi detection. Reproduced from Reference [46] with permission. Copyright: 2014 American Chemical Society.

The structure of human body is very complex and delicate, and the tumor microenvironment is intricate. We still do not have a comprehensive understanding of the tumor immune cycle, including immune checkpoints. However, with progressing research, we are trying to develop drugs with more powerful functions and better biocompatibility. For peptides, how to screen suitable candidates accurately and quickly with high affinity and specificity from the ligand library is an urgent problem to be solved. Traditional combinatorial chemical screening methods and phage display methods have long cycles, low efficiency, and make it difficult to insert or modify unnatural amino acids, which are all great limitations. Developing high-throughput methods to quickly complete screening is a promising strategy. Wang et al. developed a series of high-throughput screening platforms based on microfluidic chips [45,46,47]. Generally, traditional combinatorial library screening methods include four steps: manual separation, identification, resynthesis, and affinity detection, which is laborious and inefficient. For the design mode, they focused on the entire screening process including in situ detection, not just synthesis and sorting, to improve the screening speed; for the design concept, the microfluidic chip was dedicated to not only screening fast but also screening well, which required the introduction of more parameters in the screening process. In order to remove the obstacles to the main rate-limiting step of mass-spectrometry sequencing, an integrated screening microarray was developed. This microarray had a sheath flow configuration; the beads that had no interaction with the target protein were eliminated under the action of a magnetic field, and the positive peptide beads were trapped in a one-well−one-bead manner and cleaved in situ through a photocleavable linker. The silicon chip was then inserted into a modified MALDI target for in situ single-bead analysis. Furthermore, a part of the photocleaved peptide was transferred to the SPR chip in a “imprinting” way for high-throughput affinity analysis of the peptide microarray, which can achieve real-time, online, label-free detection, and innovatively realize the simultaneous qualitative (in situ sequencing) and quantitative (in situ affinity characterization) analysis of two sets of arrays (Figure 7). Additionally, the degenerate peptide library is also an effective approach to achieve efficient screening. Chevalier et al. invented a computer modeling platform called “Rosetta” which can reduce the astronomical total peptide library capacity to 22,660, and designed thousands of unnatural source peptides with a length of about 40 amino acids [48]. The Rosetta modeling software predicted that these peptides can not only bind tightly to the molecular target, but also inhibit the normal function of the target protein, providing the possibility for rapid peptide screening.

**Figure 8:**
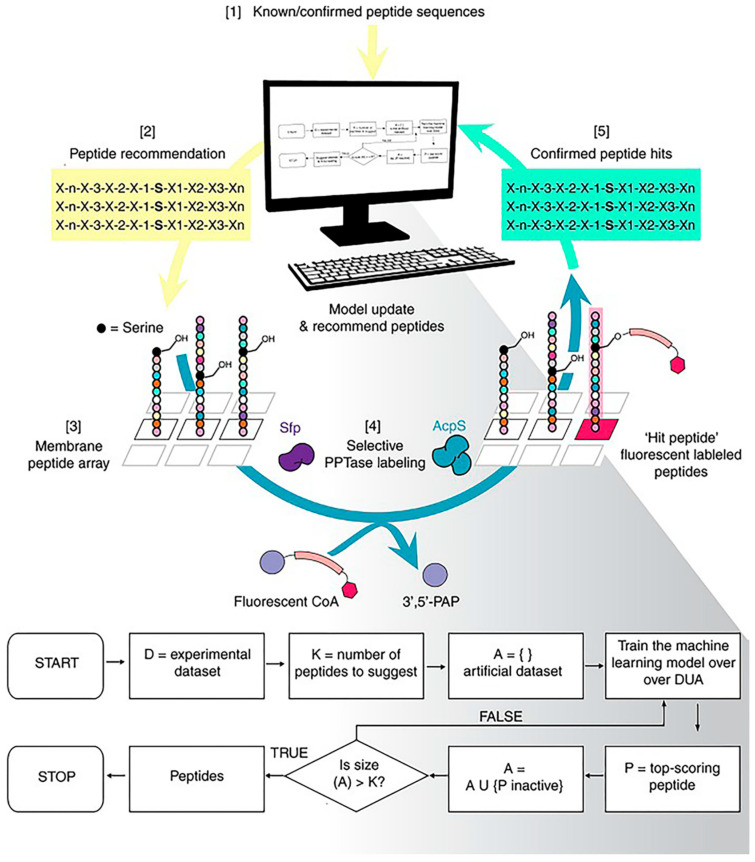
Machine-learning-based strategies for peptide screening. Using the peptide optimization with optimal learning (POOL) method to discover orthogonal peptide substrates for 4′-phosphopantetheinyl transferase. Reproduced from Reference [50] with permission. Copyright: 2017 Springer Nature.

Normally, the peptides we screen have an unsatisfactory intake caused by the obstruction of the immune system and the elimination of blood circulation. One of the reasons for this inefficiency is that we ignore the complex biological environment during the screening process. Frederixet al. demonstrated computational tools to identify the self-assembly tendencies of all possible tripeptide combinations in 20 amino acids [49]. Subsequently, by comparing with the known examples, a set of self-assembled peptide design rules was obtained and, based on this, the first tripeptide capable of forming a hydrogel at neutral pH was screened. Tallorin et al. used an optimal learning approach, Bayesian optimization, to develop a method entitled peptide optimization with optimal learning (POOL) to identify short peptides as selective substrates for enzymes (Figure 8) [50]. They demonstrated that POOL can guide the evolution of optimized orthogonal peptide substrates by alternating between prediction and targeted experimentation. This targeted approach is a departure from methods that randomly generate and experimentally screen many peptides, and can optimize for multiple complex biochemical activities, such as the ability to be selective for specific enzymes and undergo chemical transformations that would normally require several tandem screens to achieve.

As mentioned earlier, peptide supramolecules have great advantages for biomedicine. However, their development has been greatly restricted due to the lack of both approaches to predict their assembly behaviors and large-scale screening methods. In order to change this situation, many studies using molecular dynamics to simulate the self-assembly behavior of peptides have been reported. For instance, Lee et al. used coarse-grained molecular dynamics to model peptide amphiphiles’ assembly process, from a spherical shape through the hydrophobic tail gathering to cylindrical fibers when tail interactions merged [51]. However, the accuracy of molecular dynamic simulations is questionable. With the advent of the era of big data, advanced technologies based on computer science such as machine learning have become a focus and introduced innovative research methods in many fields. Li et al. used machine learning to design a program for screening the self-assembly behaviors of dipeptide hydrogels [52]. This outstanding work mainly consisted of two parts: The first was using combinatorial chemistry to build a library with a capacity of 2304 peptides synthesized from 31 monomers. The second was that 7,163,136 effective structural parameters were obtained according to their calculations, and machine-learning methods were then employed to calculate the relationships between the data points, which can help to predict the formation of unknown hydrogels and to develop reasonable designs. For life sciences, learning from nature, screening for targets, and applying concepts to human body not only provide a shortcut for us to learn the profound life sciences, but also provide strong support for our research.

## 5. Conclusions and Prospects

Peptides with high affinity and specificity can be screened out through efficient screening methods and then, after rational design, peptide-based nanomaterials with composite functions are obtained. Whether they are used for immune checkpoint blockade with enhanced blocking effect or as drug carriers for combination therapy, these nanomaterials have shown great clinical transformation potential, which is expected to bring new breakthroughs for tumor immunotherapy. For peptide-based drugs in tumor immunotherapy, some peptide tumor vaccines have entered clinical trials, but for immune checkpoint blockade, low affinity and low stability are still the main factors limiting peptide-based inhibitors’ translation into the clinical setting [53,54]. The introduction of nanomaterials may address these issues to a certain extent. However, there are still many problems to be solved. Firstly, these nanomaterials can form into delicate structures in solution only with precise control of external factors such as concentration and acidity, which cannot be achieved in the complex organisms. On the one hand, the screening and designing processes are relatively simple, but these ignore the complexity and dynamics of the living environment. On the other hand, our understanding of the human body is not deep enough at this stage, and the behavior of nanomaterials after entering the blood circulation is not yet fully trackable. All these issues highlight the necessity of developing new research methods such as machine learning. Secondly, compared with other targets, immune checkpoints involve complex physiological processes and related signaling pathways, as well as multiple organs and multiple system linkages, which require more stable, long-lasting, and controllable blocking reagents. For peptide-based nanomaterials, the introduction of some unnatural molecules, such as D-type amino acids or polymers, has been proven to be an effective method, but it also brings about the problems of highly toxic side effects and low bioavailability. With the development of chemistry, medicine, and other related fields, these problems may be alleviated, or new solutions for improving the properties of these nanomaterials may be proposed directly. In brief, peptides as endogenous substances have broad application prospects in tumor immunotherapy, and developing new screening methods as well as constructing reasonable functional molecules are effective approaches.
